# Transcriptomics and systems biology identify non-antibiotic drugs for the treatment of ocular bacterial infection

**DOI:** 10.1016/j.isci.2022.104862

**Published:** 2022-08-02

**Authors:** Susmita Das, Sukhvinder Singh, Sarthak Satpathy, Manoj Bhasin, Ashok Kumar

**Affiliations:** 1 Department of Ophthalmology, Visual and Anatomical Sciences, Kresge Eye Institute, Wayne State University School of Medicine, Detroit, MI, USA; 2 Department of Biomedical Informatics, Emory University, Atlanta, GA, USA; 3 Department of Pediatrics, Emory University, Atlanta, GA, USA; 4 Department of Biochemistry, Microbiology, and Immunology, Wayne State University School of Medicine, Detroit, MI, USA

**Keywords:** Medicine, microbiology, applied microbiology

## Abstract

Increasing antibiotic resistance among ocular pathogens often results in treatment failure for blinding infections such as endophthalmitis. Hence, newer therapeutics is needed to combat multidrug-resistant infections. Here, we show a drug repurposing approach using a connectivity map based on temporal transcriptomics of *Staphylococcus aureus* (SA) infected mouse retina. The analysis predicted three non-antibiotic drugs, Dequalinium chloride (DC), Clofilium tosylate (CT), and Glybenclamide (Glb) which reversed the SA infection signatures. Predicted drugs exhibited anti-inflammatory properties in human retinal cells against sensitive and resistant strains of SA. Intravitreal administration of all drugs reduced intraocular inflammation in SA-infected mouse eyes while DC and CT also reduced bacterial burden. Drug treatment improved visual function coinciding with reduced Caspase-3 mediated retinal cell death. Importantly, all drugs exhibited synergy with vancomycin in improving disease outcomes. Overall, our study identified three non-antibiotic drugs and demonstrated their therapeutic and prophylactic efficacies in ameliorating intraocular bacterial infection.

## Highlights

• CMap analysis predicted three drugs to reverse *Staphylococcus aureus* endophthalmitis signature
• Drugs attenuated MRSA and MSSA induced inflammatory response in retinal cells
• Drug treatment ameliorated experimental *S. aureus* endophthalmitis
• Predicted drugs exhibited adjunct therapeutic potential with antibiotic

## Graphical abstract

**Figure:**
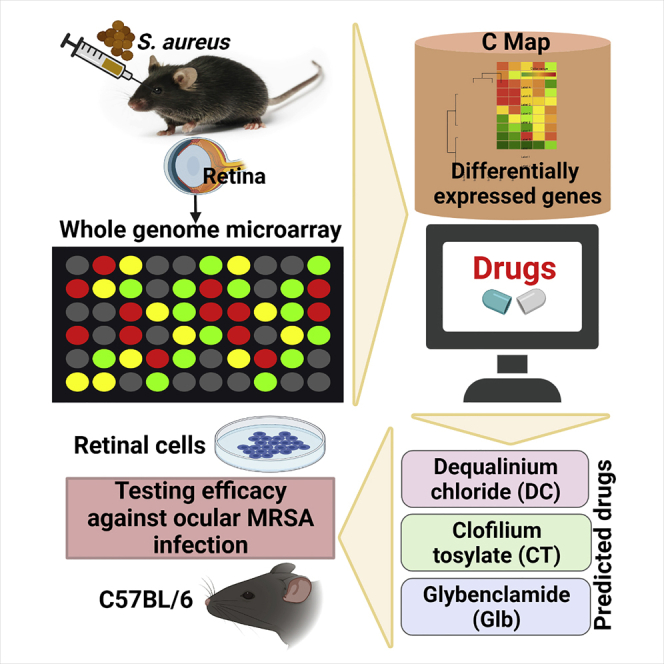

## Introduction

Bacterial endophthalmitis, one of the most significant post-operative ocular complications, is a major concern among ophthalmologists owing to its potential to cause blindness, if not treated properly (Zafar et al., 2020; Gao et al., 2020). *Staphylococcus aureus* (SA) is an important and commonly associated Gram-positive bacteria causing endophthalmitis following cataracts and other eye surgeries with poor outcomes ((Microbiologic factors and visual outcome, 1996), Lalwani et al., 2008). The increasing morbidity and mortality of staphylococcal infections are attributed to their increasing resistance to first-line antibiotics, such as methicillin and vancomycin (Rybak et al., 2013), as well as their reduced susceptibility and increased side effects to newer agents, including linezolid (Gu et al., 2013) and daptomycin (Levine, 2008). Like systemic infections, recent reports suggest that ∼50% of ocular *S. aureus* isolates from keratitis/endophthalmitis are methicillin-resistant (MRSA) and they often cause aggressive infections leading to significant vision loss (Callegan et al., 2007; Bispo et al., 2015; Pachigolla et al., 2007; Kolar and McDermott, 2011; Hernandez-Camarena et al., 2015; Hsiao et al., 2012; Deramo et al., 2008). Additionally, vancomycin-resistant SA endophthalmitis has also emerged as post-surgical complication in recent years leading to poor visual outcomes (Pathengay et al., 2011; Das et al., 2011; Relhan et al., 2015; Stroh, 2012; Gupta et al., 2011). The situation is further compounded by the fact that staphylococcal infections are challenging to eradicate owing to their ability to make biofilms on intraocular or contact lenses, which impede both the penetration of antibiotics and the infiltration of immune cells (Bispo et al., 2015; McCarthy et al., 2015; Talreja et al., 2014a, 2014b). To overcome the obstacle caused by antibiotic resistance, the discovery of newer antimicrobials continues to be an important area of investigation and drug development.

One of the ways to identify drugs/molecules with antimicrobial properties includes drug repurposing. Discovering additional uses for already approved drugs provides the quickest possible transition from bench to bedside. *De novo* drug discoveries, being a lengthy, laborious, expensive, and risky process give way to the repurposing of already known FDA-approved, discontinued, or experimental drugs in treating microbial infections (Rudrapal et al., 2020). This approach has a higher potential to be deployed over the conventional drug discovery process by alleviating the cost, longer time frame, and possibility of failure. In contrast, there is ∼45% of failure risk owing to safety or toxicity issues in the traditional drug discovery approach (Chong and Sullivan, 2007; Xue et al., 2018). Additionally, repurposing older drugs allows pharmaceutical companies to market them quickly as newer antimicrobials fail to provide financial feasibility.

In our search for alternative therapeutics to treat ocular infections, we used transcriptomics to understand the genome-level alterations involved in the host response during bacterial endophthalmitis. We performed a comprehensive temporal gene expression analysis and adopted an innovative system biology approach to identify key molecules and pathways associated with SA endophthalmitis, with most regulating inflammatory responses in this disease (Rajamani et al., 2016). Although inflammation is necessary to defend the host against invading pathogens, the eye, being an immune-privileged organ, is extremely sensitive to inflammation-mediated tissue damage if inflammation persists (Kumar et al., 2013; Kochan et al., 2012; Shamsuddin and Kumar, 2011; Kumar and Kumar, 2015). Unfortunately, monotherapy with intravitreal antibiotics remains the current standard of treatment for bacterial endophthalmitis (Callegan et al., 2007). However, the antibiotics, while destroying the bacteria, may release various bacterial cell wall components, (van Langevelde et al., 1998a, 1998b) which induce an inflammatory response, as reported in studies from our (Kumar and Kumar, 2015) and other laboratories (Heumann et al., 1994; Callegan et al., 1999). Our study involves a comparison of the genomic signatures of host cells/tissues following treatment with a drug and the incitement of disease to identify drugs that can modify the expression of innate immune markers involved in the host response.

Previously we used the connectivity map (CMap) database, a unique resource for drug repurposing, comprising >7,000 genomic profiles corresponding to 1,309 small bioactive molecules and FDA-approved drugs (Lamb et al., 2006; Lamb, 2007) and identified an anti-protozoal drug for the treatment of renal (Zerbini et al., 2014), lung cancer (Jahchan et al., 2013), and inflammatory bowel disease (IBD) (Dudley et al., 2011). This prompted us to perform CMap analysis to rapidly identify pre-approved drugs/molecules which could reverse genes/pathways dysregulated during bacterial endophthalmitis. Here, we report the therapeutic efficacy of three predicted drugs using *in vitro* and *in vivo* models of SA ocular infections.

## Results

### CMap analysis identified repurposed drugs to treat *Staphylococcus aureus* endophthalmitis

**Figure 1:**
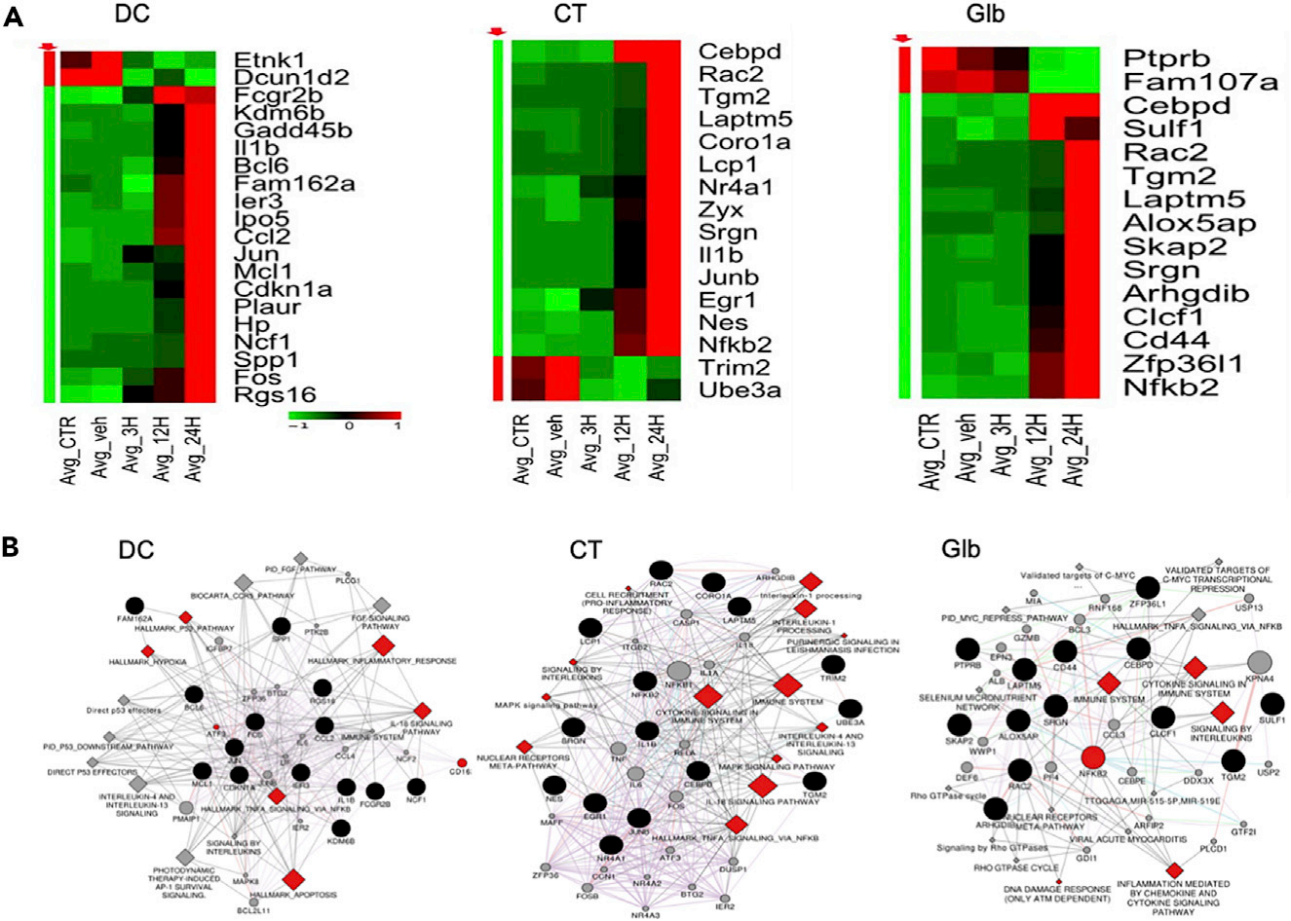
Heat maps and network analyses showing the interaction of drugs with SA infection signatures (A) The top three drugs, dequalinium chloride (DC), clofilium tosylate (CT), and glibenclamide (Glb) maximum counter regulated *S. aureus* (SA) infection signature during endophthalmitis in mice. Selected set of genes reversed by DC, CT, and Glb. Green and Red colors represent down- and up-regulation of genes, respectively. Rows represent genes and columns represent uninfected and SA-infected samples (3h, 12h, &24h). (B) Top differentially expressed (DE) genes from the drug signatures were analyzed using the GeneMANIA [version: 3.3.5] plugin in Cytoscape [Version: 3.9.0] and the gene interaction networks were built. The DE genes from the heatmaps are indicated in black and the related genes/pathways from GeneMania are represented in gray. The DE genes and related genes are represented by circles and related pathways, or gene sets are illustrated by diamonds. The red highlighted symbols represent specific genes/pathways significantly contributing to SA endophthalmitis.

Previously, we performed temporal transcriptomic analyses of retinal tissue from SA-infected mouse eyes and determined differential gene expression during bacterial endophthalmitis (Rajamani et al., 2016). Here, we used the transcriptomics data and performed systems biology approach in combination with CMap to predict the potential repurposed drug candidates. Briefly, from the gene expression profiles of temporal *SA-*infected mouse retinal samples, we developed a signature set of the top 200 up-regulated and down-regulated genes that distinguish the infected samples from uninfected controls. This signature set was then used to identify candidate drugs from the CMap database that reversed the expression of infection signature, i.e. those which counter-regulate the infection-associated genes and/or pathways. Using this approach, we identified the top three drug candidates, dequalinium chloride (DC), clofilium tosylate (CT), and glibenclamide (Glb). The predicted drugs restored the expression of master regulators perturbed in *SA* endophthalmitis (Figure 1A). Besides, the advanced network analysis revealed that the candidate drugs primarily regulated genes/pathways involved in inflammatory response (e.g., cytokine, interleukin signaling, cell death) and antimicrobial activity (Figure 1B). These predicted drugs either in combination or alone were evaluated for their therapeutic efficacy in *SA in vitro* and *in vivo* endophthalmitis experimental models.

### Identified drugs did not cause cellular toxicity at a desired dose of treatment

**Figure 2:**
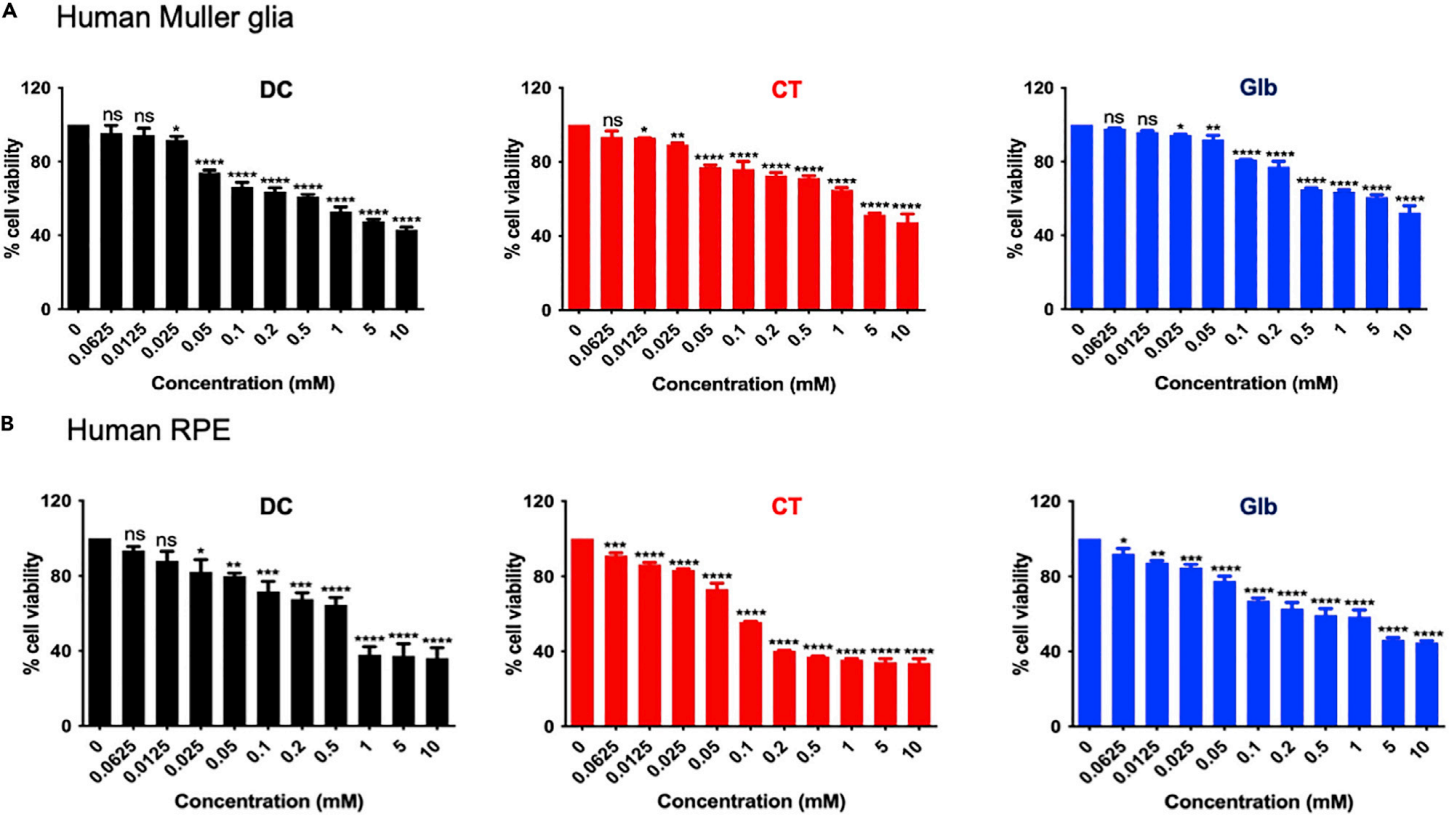
Cytotoxic effects of predicated drugs on cultured retinal cells (A) Human retinal Müller glia (MIO-M1 cell line) and (B) Human retinal RPE (ARPE-19 cell line) cells were seeded in a 96-well plate followed by exposure to dequalinium chloride (DC), clofilium tosylate (CT), and glibenclamide (Glb) at different concentrations for 16h. The cells were rinsed with 1X PBS and replaced with fresh medium. MTT assay was performed on the cells and expressed as cell viability (%) compared to control untreated cells. Data are represented as mean ± SD. Statistical analysis was performed using ANOVA(∗) p < 0.05 (∗∗) p < 0.01 (∗∗∗) p < 0.001 (∗∗∗∗) p < 0.0001. The experiment was performed twice with three biological replicates.

Current treatment options for bacterial endophthalmitis involve intravitreal injections of antibiotics. Once inside the eye these drugs interact with multiple retinal cell types, including retinal pigment epithelium (RPE) and Müller glia. Therefore, we performed *in vitro* drug cytotoxicity experiments using these cell types. Cultures of human Müller glial (MIO-M1) and human RPE (ARPE-19) cell lines were exposed to varying concentrations (0.0625, 0.0125, 0.025, 0.05, 0.1, 0.2, 0.5, 1, 5, and 10 mM) of all three drugs. After 24h, cell viability was evaluated by MTT assay. We observed that all three drugs did not exert significant cellular toxicity at lower concentrations. However, treatments with higher conc. (5 or 10mM) resulted in significant cell death in both cell types. Therefore, we selected the intermediate concentrations for all drugs DC (25μM), CT (25μM), and Glb (50μM) showing ∼80-90% cell viability in both Müller glia (Figure 2A) and RPE cells (Figure 2B).

### Predicted drugs exert anti-inflammatory properties in *Staphylococcus aureus*-infected retinal cells

**Figure 3:**
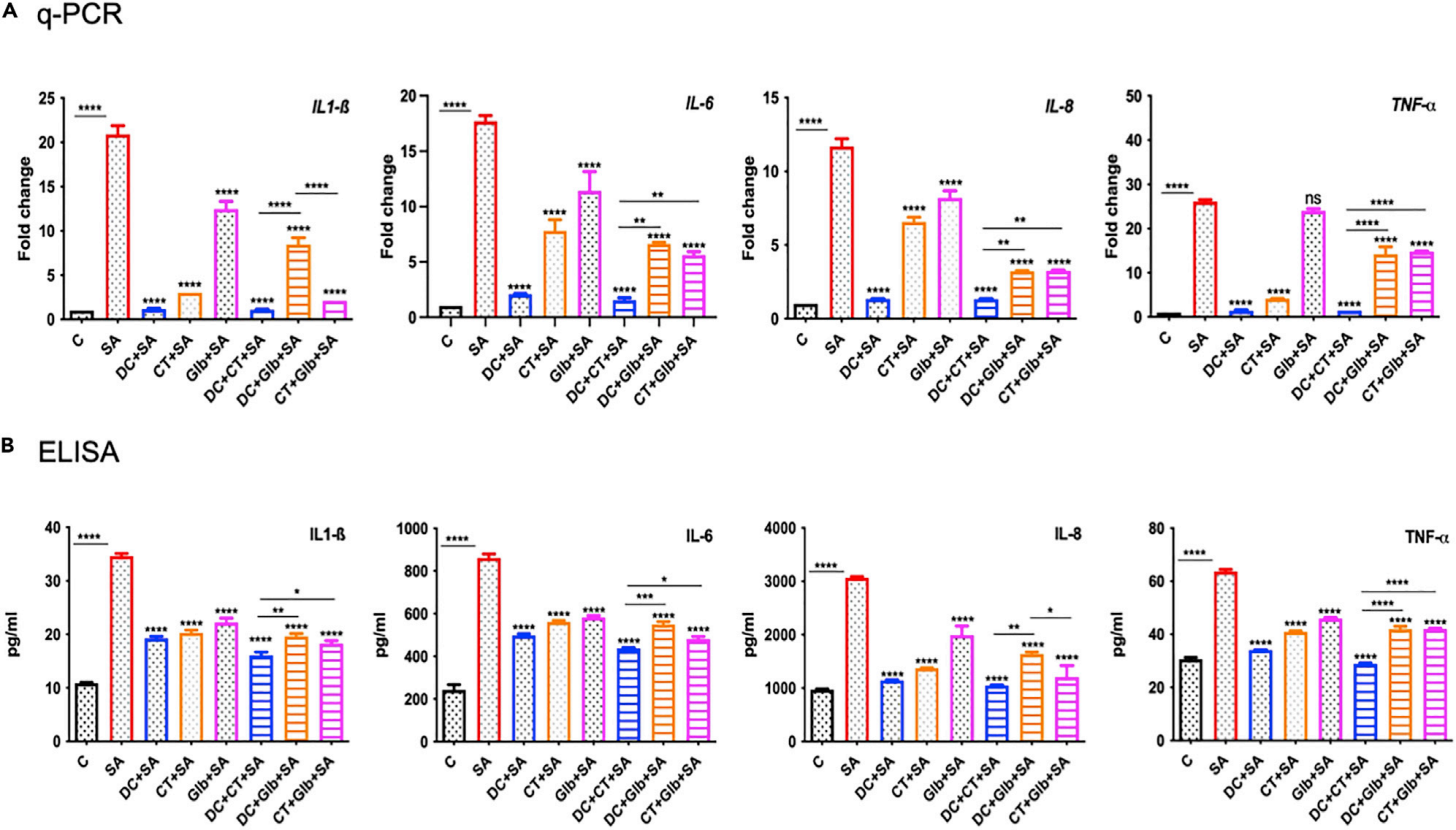
Effect of drug treatment on *S. aureus* induced inflammatory response in retinal Müller glia Human retinal Müller glial cells (MIO-M1 cell line) were treated with DC (25μM), CT (25μM), and Glb (50μM) for 1 h, followed by infection with methicillin-sensitive *S. aureus* strain RN6390 (MOI 10:1) for 6 h. Cells were harvested for qPCR analysis of inflammatory cytokines (A) and culture supernatants were used to quantify the protein levels by ELISA (B). Data are represented as mean ± SD. Statistical analysis was performed using ANOVA(∗∗∗∗) p < 0.0001 ns, non-significant. Significance was compared between uninfected control, C vs SA and SA vs drug-treated samples. The results are cumulative of two independent experiments. See also Figure S1 and S2.

Because CMap and advanced network analysis revealed that the predicted drugs modulate cytokine signaling, we assessed their effects in response to SA infection. Cultured human retinal Müller glia and RPE cells were pre-treated with all three drugs alone or in combinations prior to SA challenge. As anticipated, SA induced the expression of various inflammatory cytokines (IL-1β, IL-6, IL-8, and TNF-α) at both mRNA (Figure 3A) and protein levels (Figure 3B) in Müller glia. However, this response was muted in cells treated with all three drugs with some differences. DC and CT alone were more effective than Glb alone with >10-fold reduction in all the cytokines levels for DC-treated cells. To determine potential synergy, drugs were also used in combinations and were found that DC and CT together showed significant suppression of inflammatory mediators as compared to DC + Glb or CT + Glb.

**Figure 4:**
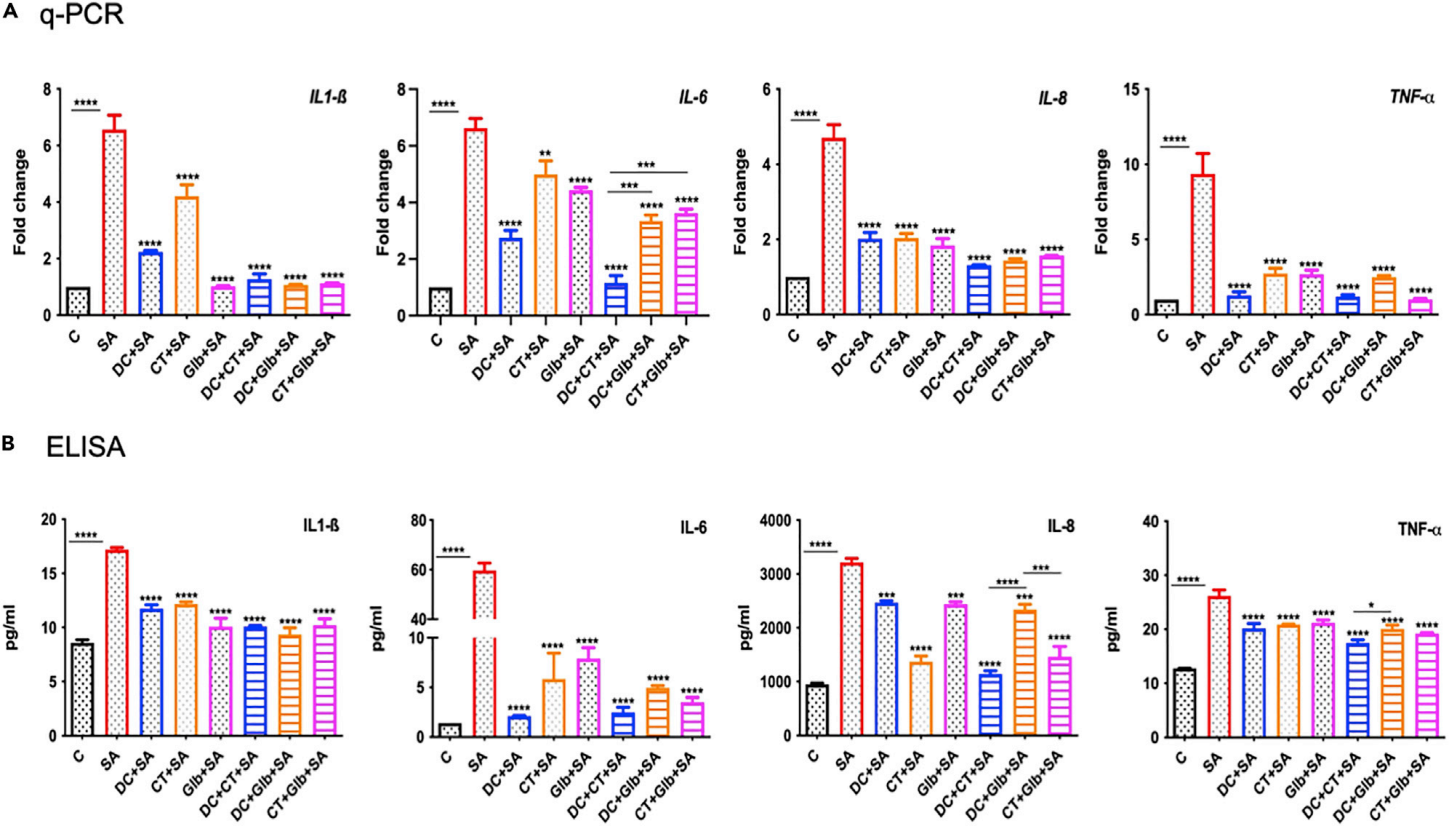
Effect of drug treatment on *S. aureus* induced inflammatory response in retinal pigment epithelial cells Human retinal pigment epithelial cells (ARPE-19 cell line) were treated with DC (25μM), CT (25μM), and Glb (50μM) for 1 h, followed by infection with methicillin-sensitive *S. aureus* strain RN6390 (MOI 10:1) for 6 h. Cells were harvested for qPCR analysis of inflammatory cytokines (A) and culture supernatants were used to quantify the protein levels by ELISA (B). Data are represented as mean ± SD. Statistical analysis was performed using ANOVA(∗) p < 0.05 (∗∗) p < 0.01 (∗∗∗∗) p < 0.0001. Comparisons were made between uninfected control, C vs SA and SA vs drug-treated samples. The results are cumulative from two independent experiments. See also Figure S3.

To determine whether the observed anti-inflammatory effects of the drugs are cell-specific, a similar experiment was performed in cultured human RPE (ARPE-19) cells. Consistent with previous results, all three drugs individually decreased mRNA (Figure 4A) and protein (Figure 4B) levels of inflammatory cytokines in SA-infected RPE cells. Overall, DC reduced levels of inflammatory mediators more than CT or Glb did alone, except for IL-1β, which was drastically reduced by Glb. But the drug combinations did not show significant differences among themselves in alleviating the cytokine levels, other than IL-8 protein levels which were significantly decreased by DC + CT treatment. Although the drugs diminished the inflammatory cytokine profile, the next aim was to find any antimicrobial properties of these identified drugs. Together, these findings show that DC, CT, and Glb treatment attenuate SA-induced inflammatory response.

### Drug treatment reduced lipopolysaccharide-induced cytokines in cultured retinal cells

As DC is a known antiseptic drug, the observed anti-inflammatory effects could be owing to its direct antibacterial activity. Therefore, we determined the MIC of all predicted drugs and observed that the MIC values of DC and CT were 12.5 and 25μM, respectively (Figure S1). In contrast, Glb did not show any inhibitory effect at all concentrations tested, the highest being 100μM. Next, we evaluated whether these drugs have direct or indirect anti-inflammatory properties using lipopolysaccharide (LPS) as stimuli. We observed that both cell lines had differential expression levels of inflammatory cytokines in response to LPS challenges. However, all three drugs (DC, CT, and Glb), significantly reduced LPS-induced mRNA and protein levels of inflammatory cytokines IL-1β, IL-8, and TNF-α, but had minimal effect on IL-6 levels in both Müller glia (Figure S2) and RPE cells (Figure S3). Also, it is pertinent to note that Glb alone or in combinations was found to exert higher anti-inflammatory activity than individual or combined DC and CT treatments. These results indicate that the predicted drugs exert direct anti-inflammatory properties.

### Identified drugs attenuate methicillin-resistant *Staphylococcus aureus* (USA300) induced inflammatory response

**Figure 5:**
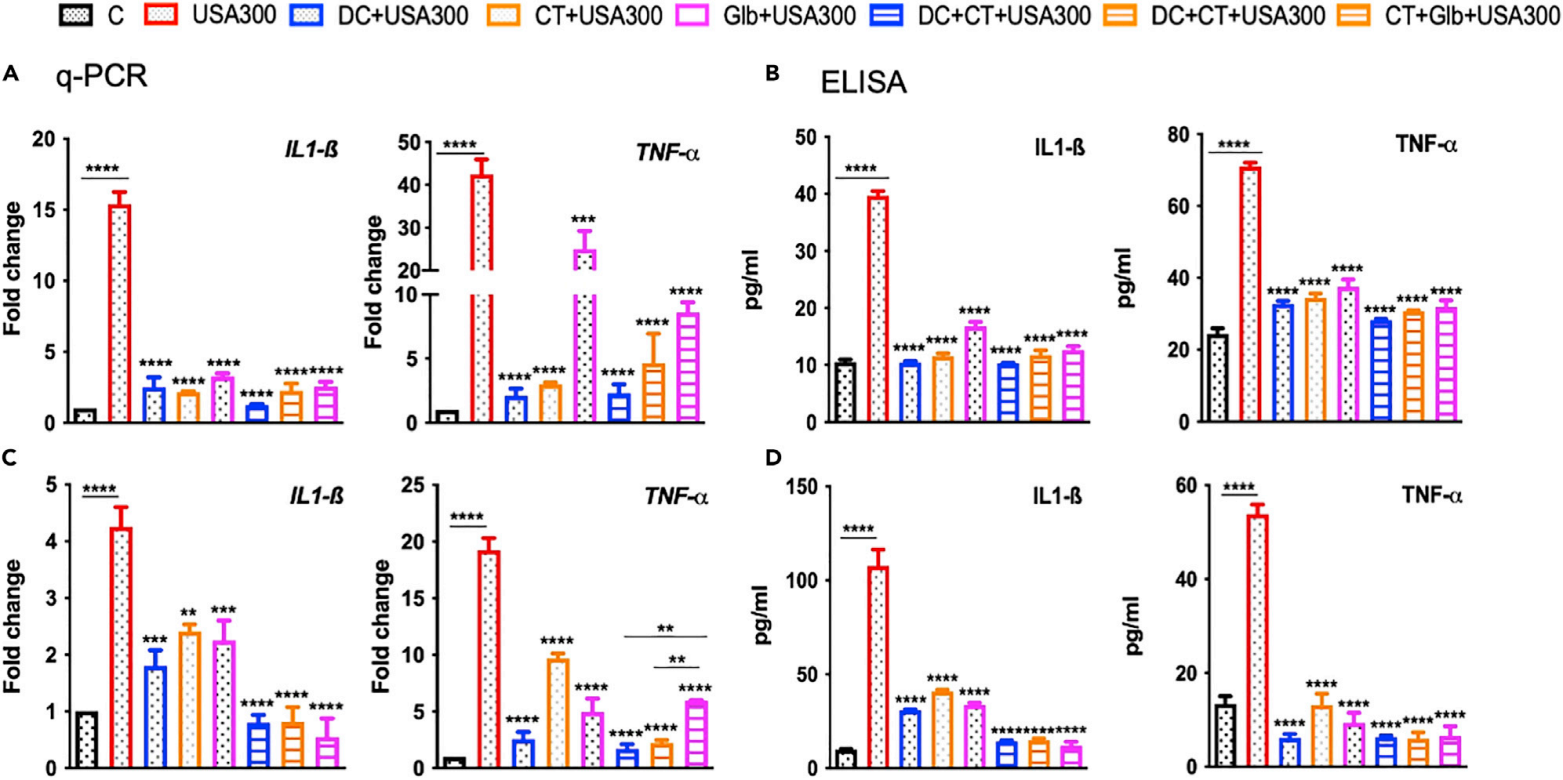
Anti-inflammatory effects of predicted drugs in MRSA-infected retinal cells (A and B) Human retinal Müller glial cells (MIO-M1 cell line) and (C-D) human retinal pigment epithelial cells (ARPE-19 cell line) were treated with DC (25μM), CT (25μM), and Glb (50μM) for 1 h, followed by infection with methicillin-resistant *S. aureus* strain USA300 (MOI 10:1) for 6 h. Cells were harvested for qPCR analysis of inflammatory cytokines (A, C) and culture supernatants were used to quantify the protein levels by ELISA (B, D). Data are represented as mean ± SD. Statistical analysis was performed using ANOVA(∗) p < 0.05 (∗∗) p < 0.01 (∗∗∗) p < 0.001 (∗∗∗∗) p < 0.0001. Comparisons were made between uninfected control, C vs SA and SA vs drug-treated samples. The results are cumulative from two independent experiments. See also Figure S4.

The goal of our study was to repurpose the drugs to treat drug-resistant ocular bacterial infections. As USA300 (MRSA strain) is frequently associated with resistant SA infections, including those in the eye, we assessed the efficacy of all three drugs against USA300 infection of retinal cells. First, we noticed that USA300 induced higher inflammatory response as compared to MSSA (RN6390 strain) in both retinal cell types. Interestingly, all the drugs drastically reduced the inflammatory cytokine IL-1β and TNF-α (data not shown for IL-6 and IL-8) in retinal Müller glia (Figure 5A) and RPE cells (Figure 5B). Drug combinations also showed a significant decrease in cytokine secretion with DC + CT and DC + Glb combinations being better than CT + Glb. Similar to live USA300, all three drugs attenuated inflammatory response incited by heat-killed USA300 (Figure S4). Overall, these results demonstrate that the identified drugs can reduce inflammation during multidrug-resistant bacterial infection.

### Drug treatment ameliorates USA300-induced endophthalmitis in mice

**Figure 6:**
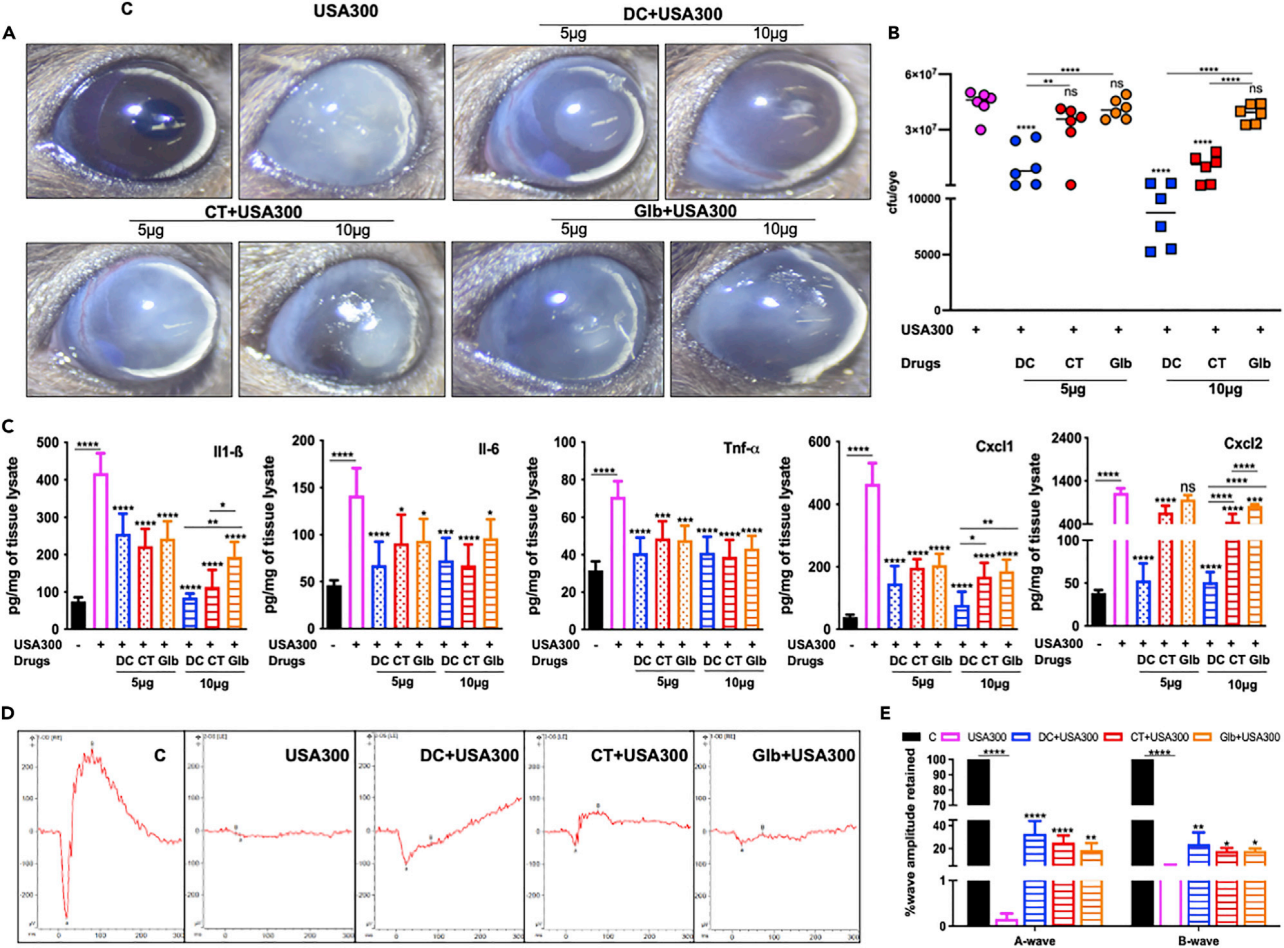
Therapeutic efficacy of predicted drugs in a mouse model of MRSA endophthalmitis C57BL/6 mice were intravitreally injected (n = 6 eyes) with 5000 colony forming units (CFU/eye) of *S. aureus* USA300 or PBS (control, C), followed by intravitreal drug injections (5μg or 10μg per eye) at 6h post bacterial infection. Eyes were analyzed 24h post-drug administration. (A) Slit-lamp examination was performed, and photomicrographs were taken from representative eyes showing corneal haze/opacity. (B) Eyes were enucleated, homogenized, and the bacterial burden was estimated via serial dilution plating. (C) The lysates from infected, drug-treated, and control eyes were subjected to ELISA to quantify inflammatory mediators. (D) Scotopic electroretinogram (ERG) analysis was performed to assess retinal function by measuring a- and b-wave amplitudes post drug treatment (10μg/eye dose). (E) Bar graph showing percent a- and b-wave amplitude retained with respect to control eyes set at 100%. Data are represented as mean ± SD. Statistical analysis was performed using ANOVA(∗) p < 0.05 (∗∗) p < 0.01 (∗∗∗) p < 0.001 (∗∗∗∗) p < 0.0001, ns; non-significant. Comparisons were made between uninfected control, C vs SA and SA vs drug-treated samples. See also Figure S5.

The *in vitro* studies demonstrated the ability of predicted drugs to reduce inflammation in response to both sensitive and resistant strains of SA. Next, we sought to determine their efficacy *in vivo* using a mouse model of USA300-induced endophthalmitis. First, a dose-dependent study was performed by using two different doses (5 and 10 μg per eye) of each drug administered intravitreally at 6h post bacterial challenge. The microscopic eye exam revealed that USA300 infection significantly increased corneal haze, opacity, and hypopyon formation. However, eyes treated with all drugs markedly reduced these pathological changes in a dose-dependent manner. Among the drugs, DC-treated eyes had lower corneal and anterior chamber opacity than CT and Glb (Figure 6A). As expected, the control eyes injected with PBS did not show any bacterial growth, whereas an average of 4.5 × 10^7^ CFU was recovered from the infected but untreated eyes. Interestingly, the bacterial burden was substantially reduced with DC administration in a dose-dependent manner. On the other hand, CT significantly reduced the bacterial density at a higher dose of 10 μg and Glb did not significantly affect the bacterial burden (Figure 6B). The ELISA assay of whole eye lysates showed a significant reduction in levels of inflammatory cytokines (IL1-β, IL-6, and TNF-α) and chemokines (CXCL-1 and CXCL-2) in the treatment group (Figure 6C). The comparative analysis indicates that overall DC was superior to CT and Glb in reducing bacterial haze, bacterial burden, and inflammatory mediators in mouse eyes. To determine whether the improved clinical presentation translates into preserved retinal function, electroretinogram (ERG) study was performed. Our data showed that all drug-treated eyes had better retention of both A- and B-wave amplitudes (Figures 6D and 6E) otherwise drastically reduced in USA300 infected eyes. Again, the DC treatment was found to be relatively better in preserving retinal function than CT or Glb.

**Figure 7:**
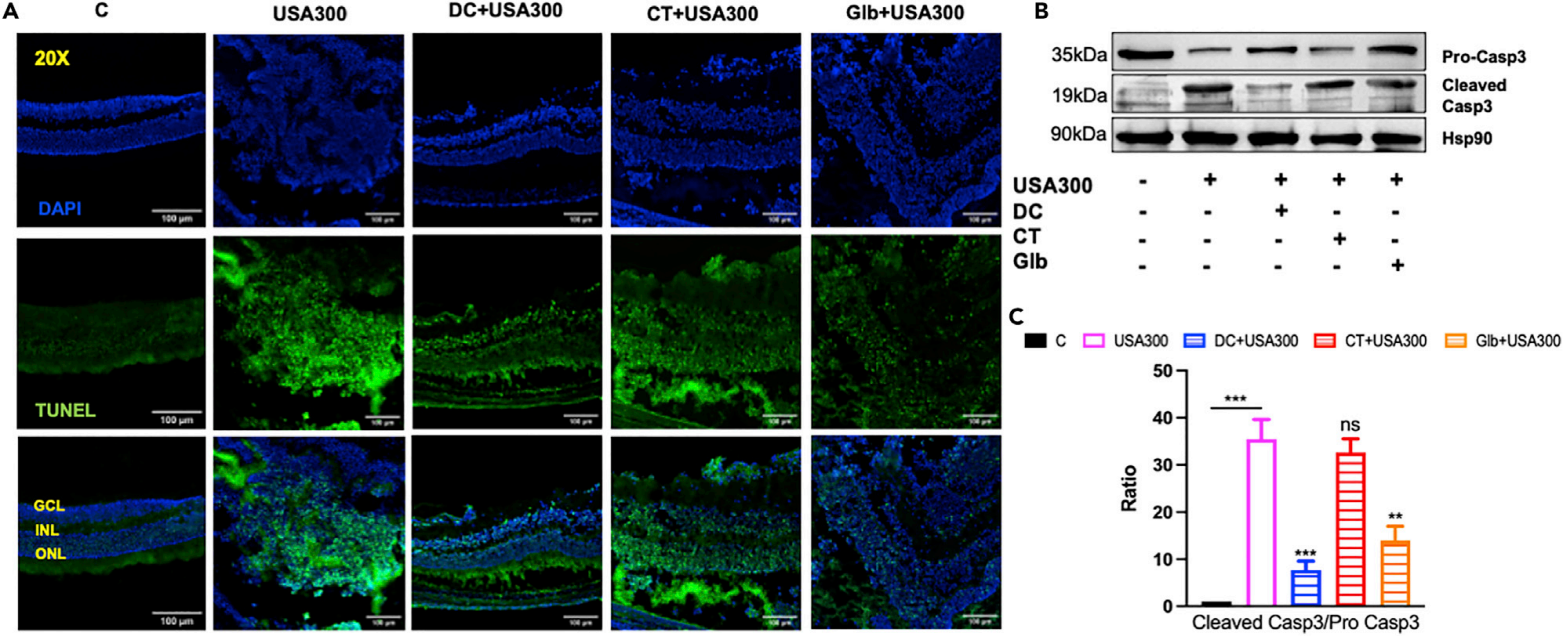
Impact of drug treatment on retinal cell death during MRSA endophthalmitis C57BL/6 mice were intravitreally injected (n = 4 eyes) with 5000 colony forming units (CFU/eye) of *S. aureus* USA300 or PBS (control, C), followed by intravitreal drug injections (10μg per eye) at 6 h post bacterial infection. (A) At 24h post-drug administration, eyes were embedded in OCT and 5-micron cryo-sections were subjected to TUNEL staining (blue, DAPI nuclear stain; green, TUNEL+ve cells), Scale bar; 100μm. (B) Western blot was performed using mouse retinal lysates to detect Pro-Caspase 3 and cleaved Caspase 3 proteins. (C) Densitometric analysis was performed using ImageStudio and expressed as relative fold changes normalized to the respective loading control, Hsp90. Data are represented as mean ± SD. Statistical analysis was performed using ANOVA(∗) p < 0.05 (∗∗) p < 0.01 (∗∗∗) p < 0.001, ns; non-significant. Significance was compared between control, C vs SA samples and SA vs drug-treated samples.

One of the potential mechanisms for reduced retinal function in SA endophthalmitis is increased retinal cell death. To assess the effect of predicted drugs on retinal cell death phenomena, we performed TUNEL assay using retinal cryosections and observed that USA300 infection disrupted retinal architecture and significantly increased TUNEL positive cells. In contrast, eyes treated with all three drugs, reduced apoptotic cells as evidenced by the decrease in both the number and intensity of TUNEL positivity (Figure 7A). Among all three drugs, DC was found to reduce retinal tissue damage better than Glb followed by CT. The effect of the drugs on cell death was further confirmed by Western blots of retinal lysates using Caspase-3 antibody (Figure 7B). Our data showed increased levels of cleaved Casp-3 in USA300 infected retinal tissue but a significant reduction in those treated with DC. Glb treatment also reduced cleaved Casp-3 but CT seemed to have a minimal effect (Figures 7B and 7C).

In addition to the therapeutic approach, we performed a prophylactic study where 10μg of each drug was administered 12h prior to USA300 infection. We observed that both DC and Glb when given prophylactically, significantly reduced corneal haze and anterior chamber opacity (Figure S5A). Although Glb and CT did not show any remarkable effect on bacterial growth as compared to their post-treatment (data not shown), DC pretreatment drastically reduced the bacterial burden (Figure S5B). The assessment of inflammatory response showed that both DC and Glb treatment markedly reduced cytokine levels. CT did not exert any significant effect on inflammatory response (data not shown) as compared to its post-treatment. Moreover, a head-on comparison showed relatively better anti-inflammatory properties of DC and Glb in prophylactic versus therapeutic approach (Figure S5C). Collectively, these results indicate that the predicated drugs were effective in ameliorating MRSA endophthalmitis when given prophylactically or therapeutically.

### Predicted drugs synergize with vancomycin to reduce endophthalmitis severity

**Figure 8:**
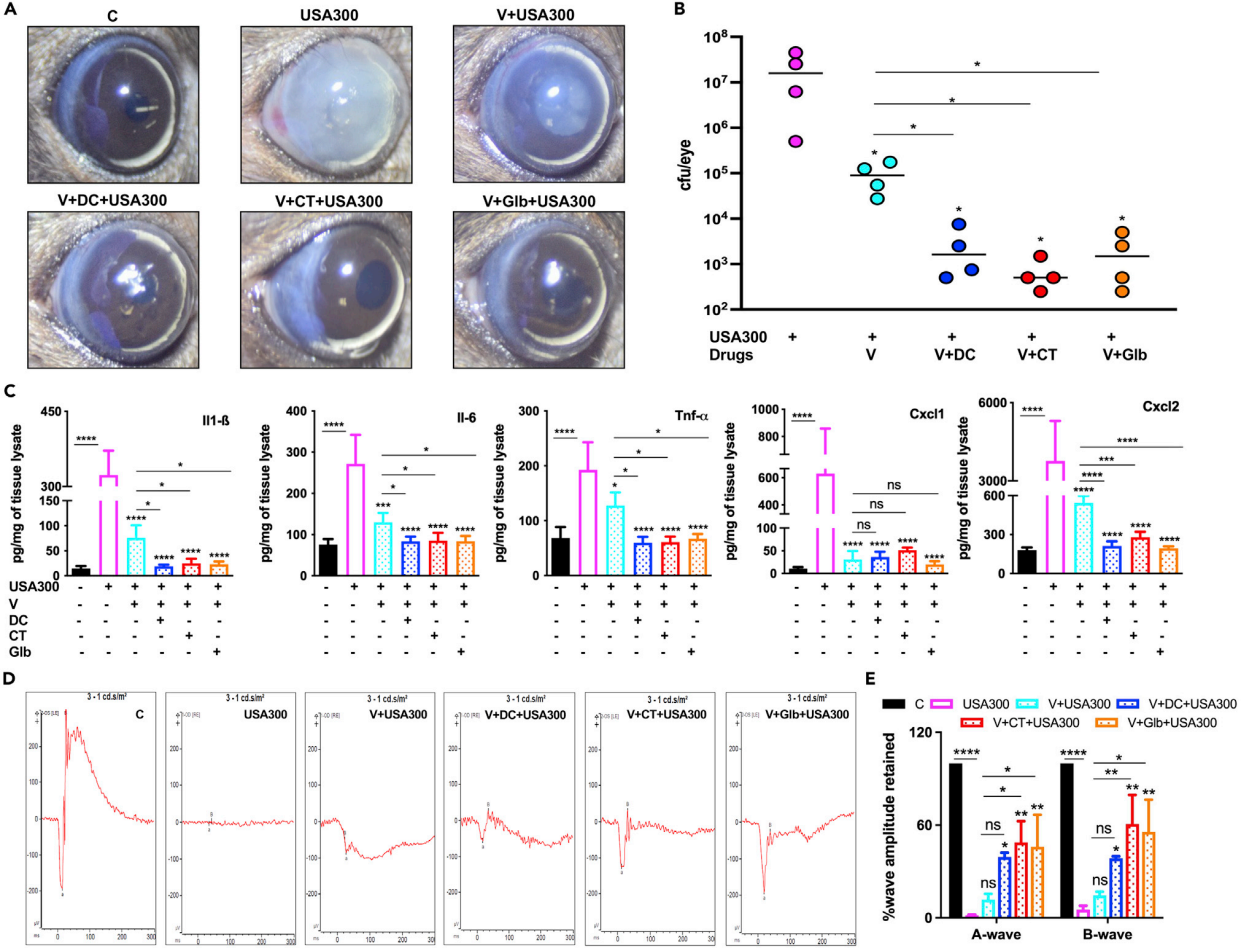
Effect of combination therapy with vancomycin on retinal cell death during MRSA endophthalmitis C57BL/6 mice were intravitreally injected (n = 4 eyes) with 5000 colony forming units (CFU/eye) of *S. aureus* USA300 or PBS (control, C), followed by intravitreal injections of vancomycin (0.7μg per eye) alone or in combination with DC, CT, or Glb (10μg per eye) at 6 h post-infection. Eyes were analyzed 24h post-drug administration. (A) Slit-lamp examination was performed, and photomicrographs were taken from representative eyes showing corneal haze/opacity. (B) Eyes were enucleated, homogenized, and the bacterial burden was estimated via serial dilution plating. (C) The lysates from control, infected, and drug-treated eyes were subjected to ELISA to quantify inflammatory mediators. (D) Scotopic electroretinogram (ERG) analysis was performed to assess retinal function. (E) Bar graph showing percent a- and b-wave amplitude retained with respect to control eyes set at 100%. Data are represented as mean ± SD. Statistical analysis was performed using ANOVA(∗) p < 0.05 (∗∗) p < 0.01 (∗∗∗) p < 0.001 (∗∗∗∗) p < 0.0001, ns; non-significant. Comparisons were made between uninfected control, C vs SA and SA vs drug-treated samples.

Intravitreal injections of vancomycin alone or in combination with ceftazidime remain the standard therapy to treat bacterial endophthalmitis (Roth and Flynn, 1997). Given the anti-inflammatory properties of the predicted drugs, we postulated whether the predicted drugs can be given as adjunct therapeutics along with vancomycin to ameliorate SA endophthalmitis. Mice were injected with a sub-MIC dose of vancomycin (Wang et al., 2006; Holmes and Jorgensen, 2008) either alone (0.7μg per eye) or in combination with DC, CT or Glb (10μg per eye). As expected, 24h post-treatment, vancomycin (V) significantly reduced both corneal haze (Figure 8A) and bacterial load (Figure 8B). Interestingly, all combination therapies further reduced bacterial burden and improved corneal transparency, indicating synergistic properties. Similarly, both vancomycin and drug combinations significantly reduced inflammatory mediators. However, the effects were profound in eyes treated with combination therapies, as evidenced by reduced levels of IL-1β, IL-6, TNF-α, and CXCL2 (Figure 8C). The combination therapy of vancomycin with DC, CT, and Glb retained retinal function in infected mice eyes as observed by the rescue in the a- and b-wave amplitudes when compared to vancomycin treatment alone (Figures 8D and 8E).

## Discussion

Healthcare-associated infections, especially those caused by multidrug-resistant Gram-positive bacteria, such as methicillin-resistant *S. aureus* (MRSA), are a growing public health threat (Roberts et al., 2015; Stryjewski and Corey, 2014). Additionally, various strains of Staphylococci have been associated with severe bacterial infections, including endophthalmitis which occasionally leads to vision loss, if not treated promptly. Post-operative endophthalmitis is one of the most common complications after cataract surgery. During the initial diagnosis of endophthalmitis and the pathogen type is unknown, the choice of antimicrobials plays an important role. Unfortunately, the clinical symptoms often fail to match with actual results (Johnson et al., 1997). Moreover, owing to delayed diagnosis and despite the administration of broad-spectrum antibiotics, visual imparity remains a potential threat ((Results of the Endophthalmitis Vitrectomy Study, 1995), Okhravi et al., 1997). The ongoing treatment for bacterial endophthalmitis majorly includes intravitreal antibiotic injections (1995, Baum et al., 1982; Brod and Flynn, 1993; Vahey and Flynn, 1991) along with a few systemic antibiotics (Aaberg et al., 1998; Romero et al., 1999), albeit reports suggesting their inability to penetrate the blood-retinal barrier and reach the vitreous (Ferencz et al., 1999). However, despite strong antibacterial activity, antibiotics such as fluoroquinolones are not recommended for direct intravitreal administration owing to possible toxic side effects (Wiechens et al., 1998; Stevens et al., 1991). Additionally, the development of vancomycin-resistant pathogens including *S. aureus* greatly impacts the therapeutic market for bacterial endophthalmitis (Callegan et al., 2002).

To find alternate therapeutic interventions, a transcriptomic study was performed to understand the gene level alterations in the host against *S. aureus* endophthalmitis in mice. A temporal gene expression analysis, together with systems biology approach identified its associated key molecules and pathways, especially those regulating inflammatory responses during the infection. Although intraocular inflammation is important for the clearance of the pathogen, it can pose a major threat to the eye tissues owing to uncontrolled inflammation. Moreover, antibiotics do not control the inflammatory responses and may interfere with visual signals. Hence, a balance of antibacterial and anti-inflammatory therapy should be considered while treating patients with endophthalmitis. As *de novo* drug discovery is both slow-paced and expensive, we used drug repurposing to find a solution to this problem. This technique exploited the connectivity map (CMap) database to counter-regulate SA endophthalmitis infection signatures with drugs that can reverse the expressions of specific genes involved in the host response. Here, we identified three drugs; DC, CT, and Glb that could reverse the expression of >60% of the master regulators perturbed in SA endophthalmitis. DC is a bis-quaternary ammonium cation with known antimicrobial activity against several Gram-positive and Gram-negative bacteria, fungi, and protozoan parasites. DC tablets (Fluomizin) have been shown to be equally as effective as vaginal clindamycin cream in the treatment of bacterial vaginosis with no systemic safety concerns (Mendling et al., 2016; Weissenbacher et al., 2012). DC has also been reported to possess anti-tumor activity and reduce the rate of metastasis by inhibiting Raf/MEK/ERK1/2 and PI3K/Akt signaling pathways (Timaner et al., 2015; Makowska et al., 2014). CT, on the other hand, is a quaternary ammonium compound that acts as an antiarrhythmic agent and a K+ channel blocker. It has been shown to have an anti-fibrillatory effect during myocardial ischemia and after infarction (Kowey et al., 1985). Glb, also known as glyburide, is an anti-diabetic drug, belonging to the sulfonylurea class of oral drugs and reduces blood glucose levels by stimulating insulin secretion (Chukwunonso Obi et al., 2016). It has been identified as the first compound to act upstream of cryopyrin to prevent PAMPs, DAMPs, and crystal-induced IL-1β secretion (Lamkanfi et al., 2009) and reduce IL-1β and IL-8 production by PMNs in cases of bacterial infections in patients with diabetes (Kewcharoenwong et al., 2013).

Drug repurposing provides several FDA-approved bioactive molecules and can be used risk-free in humans. Our study revealed that all three drugs were non-toxic at certain doses when tested on retinal cells. The eye, being an immune privilege organ with a complex retinal network, the contribution of retinal cell types in inducing innate immune responses to *S. aureus* infection post drug treatment was determined on cultured human retinal Müller glia and RPE, representing retinal residential cells (Kumar et al., 2013; Strauss, 2005; Linehan and Fitzgerald, 2015). In addition to providing structural stability to the retina, Müller glial cells maintain ion homeostasis, ammonia levels and confer protection to the blood-retinal barrier (BRB). Recent studies have proved that these cells actively mediate retinal innate immunity, especially in infectious endophthalmitis (Kumar et al., 2013). BRB, primarily comprised of the endothelial cell and a single layer of RPE cells forming the outer barriers, protects the eye from systemic circulation and infection (Singh et al., 2019). Through its contact with choroid and photoreceptors, RPE recognizes pathogenic stimuli as well as any drug molecule reaching from both choroid and vitreous. Hence, Müller glia and RPE are essential in eliciting innate immune responses via receptors activation, cytokine/chemokine secretion, and complement components during retinal infections (Detrick and Hooks, 2010; Kumar et al., 2004). As expected from our in-silico data, all three drugs significantly reduced inflammatory response during the SA challenge. Interestingly, the drug combinations showed greater impact with DC + CT being most effective in down-regulating inflammation. This observation led to the evaluation of bacterial growth inhibition in presence of these drugs. DC, already known for its anti-infective property to treat bacterial vaginosis (Bailly, 2021) and CT, reportedly showing bactericidal activity against *Acinetobacter baumannii* and *S. aureus* (Knauf et al., 2018), inhibited SA growth in our study. This led to a concern about whether the reduction of an inflammatory response by these two drugs was directly owing to microbial killing. To further evaluate this phenomenon, LPS stimuli were used to challenge the retinal cells in presence of drugs. LPS is a major virulence factor present on the cell wall of Gram-negative bacteria and its addition can potentially initiate inflammatory signaling in these cell types. Our data show that all three drugs suppressed inflammatory cytokines, except IL-6 by DC and CT. Moreover, in this case, Glb was able to drastically reduce the cytokine levels as compared to other drugs, when treated alone, while DC + Glb and CT + Glb combinations were more effective than DC + CT, indicating Glb to have a potent anti-inflammatory effect. DC and CT, on the other hand, possess both antibacterial as well as anti-inflammatory properties.

MRSA is increasingly being reported in causing ocular infections, including endophthalmitis (O'Rourke et al., 2021). Owing to the growing concern of multidrug resistance (MDR) among MRSA strains (Huz et al., 2017) and treatment failures, we tested the ability of the predicted drugs to reduce the inflammation in response to USA300 infection. Our data showing attenuated inflammatory response by all three drugs either alone or in combinations indicate the potential use of these drugs in MDR infections. The antibiotic treatment while killing the bacteria releases various bacterial cell wall components which can cause lingering inflammation in the eye. Interestingly, our experiments using heat-killed USA300 (HKU) show that the drugs are still effective in reducing inflammation against deadly bacteria. Therefore, these findings support our hypothesis that the predicted drugs work against drug-resistant strains of *S. aureus* infection through microbial killing as well as abating inflammatory response. To further support this idea, we tested the efficacy of the predicted drugs *in vivo* in a mouse model of USA300-induced endophthalmitis. First, we tested the drugs in a therapeutic manner by giving intravitreal injections 6h post-SA infection. Our dose-response study indicated that both DC and CT reduced bacterial burden where CT exerted a significant antibacterial effect at a relatively higher dose. However, Glb treatment did not reduce the bacterial burden in the eye, but remarkably attenuated SA-induced expressions of cytokines and chemokines. This indicates that Glb can be used in combination with CT and DC to have synergistic bacterial killing and anti-inflammatory effects. Indeed, both DC and CT treatments also diminished inflammatory mediators in infected mouse eyes, suggesting their therapeutic potential in treating bacterial endophthalmitis. In addition to reduced bacterial burden and inflammation, the ERG analysis showed significant retention of retinal function. DC treatment could retain the ERG response better than CT and Glb, which is likely owing to its potent antimicrobial and anti-inflammatory properties. Recently, Glb has been shown to protect mice retinal neurons in diabetic retinopathy and excitotoxicity models (Berdugo et al., 2021), which can be related to its anti-inflammatory effects. The reduced number of apoptotic retinal cells corroborated with a decreased expression of protein involved in cell death and apoptosis in the drug-treated eyes, especially with DC. Hence, the improvement in retinal function observed with drug administration could be owing to reduced retinal cell death.

Bacterial endophthalmitis is mostly associated with ocular surgeries, such as cataract. During cataract surgery, the antibiotics are infused in the anterior chamber as prophylactic approach to avoid endophthalmitis. Moreover, intravitreal injections of antibiotic and anti-inflammatory drugs prior to surgeries have also been used not only to minimize endophthalmitis cases but to reduce the frequency of pre- and post-surgical drops (Lindstrom et al., 2017; Tyson et al., 2017). One of the complications of cataract surgery is rupture of the posterior lens capsule resulting in lens-induced inflammation (Sridhar and Tripathy, 2022; Xu et al., 2011). As our predicted drugs possess anti-inflammatory properties, we postulated that drugs might be beneficial when given prophylactically. Our data showed that DC pre-treated eyes had a drastic reduction in bacterial burden, supporting the notion that drugs can be given prophylactically to prevent accidental ocular infection. Moreover, both Glb and DC pretreatment reduced the inflammatory response in SA-infected eyes, indicating their benefits as prophylactic anti-inflammatory drugs that can be used before ocular surgeries to minimize inflammation.

Current treatment for bacterial endophthalmitis involves the intravitreal injections of vancomycin and ceftazidime. Although essential to restrict bacterial growth, antibiotic-killed bacteria can still trigger an inflammatory response by activating TLRs and other PRRs in retinal cells (Das et al., 2021). Thus, adjunct anti-inflammatory therapeutics could be beneficial for the treatment of endophthalmitis. Indeed, our recent study showed the therapeutic use of cellular metabolites, itaconate in ameliorating SA endophthalmitis (Singh et al., 2021b). Using a similar approach, we tested the therapeutic efficacy of predicted drugs in combination with antibiotics. Our data showed that the predicted drugs exerted synergistic effects with vancomycin in reducing both bacterial burden and inflammation. The eyes treated with combination therapy had better disease outcomes, including improved retinal function, indicating their use as an adjunct therapy.

In conclusion, using transcriptomic data and CMap analysis, we were able to identify three potential drugs and demonstrated their therapeutic and prophylactic efficacy in ameliorating SA endophthalmitis. The predicated drugs were found to possess both antibacterial and anti-inflammatory properties and can be used alone or in combination to attenuate inflammatory responses. Most importantly, the drugs were found to be effective in reducing inflammation triggered by both MSSA and MRSA infections and can potentially be used as an adjunct therapy with lower antibiotic dosage. Collectively, our study provides a proof of concept to identify repurposed drugs and test their effectiveness in *in vitro* and *in vivo* models of bacterial infection.

### Limitations of the study

Despite the important findings in our study, a few limitations should be considered. Endophthalmitis is caused by both Gram-positive and -negative bacteria as well as fungi, while we only tested the drugs’ efficacy against *S. aureus*, a Gram-positive pathogen. Secondly, the drugs were administered 6h post-infection which limits to evaluate of their efficacy when given during the advanced disease stage, when actual symptoms develop. Therefore, additional studies are required to test these drugs against Gram-negative bacteria and to gain in-depth knowledge about the dose and timing of drug administration for real case scenarios. Although reports have shown DC to possess efficacy like clindamycin in other infection models, the drugs were not directly compared with that of other conventional antibiotics. Our study shows therapeutic and prophylactic effectivity of the drugs individually in the mouse model of endophthalmitis, but the result for combinational therapy still needs to be addressed *in vivo*. Finally, we did not fully elucidate the mechanisms underlying the antibacterial and anti-inflammatory properties of the predicted drugs.

## STAR★Methods

### Key resources table

**Table:** 

REAGENT or RESOURCE	SOURCE	IDENTIFIER
**Antibodies**		

Caspase-3 Rabbit Ab	CST	9662S; RRID: AB_331439
Hsp90 (C45G5) Rabbit mAB	CST	4877S; RRID: AB_2121214

**Chemicals**		

Dequalinium chloride	Santa Cruz Biotechnology, Inc.	sc-214869
Clofilium tosylate	Santa Cruz Biotechnology, Inc.	sc-391228
Glyburide (Glibenclamide)	Santa Cruz Biotechnology, Inc.	sc-200982
Vancomycin	Cayman Chemicals	15327
TRIzol reagent	Life technologies	15596018
FBS (Fetal bovine serum)	CPS Serum	FBS-500-HI
Dulbecco’s modified Eagle’s medium GlutaMAX	Gibco	10566016
Dulbecco’s modified Eagle’s medium Nutrient mixture F-12	Gibco	11330032
Lipopolysachharide (LPS-B5)	InvivoGen	055:B5

**Critical Commercial Assays**		

Mouse IL-1β ELISA kit	R&D System	DY401
Mouse IL-6 ELISA kit	R&D System	DY406
Mouse TNF-α ELISA kit	R&D System	DY410
Mouse CXCL-1 ELISA kit	R&D System	DY453
Mouse CXCL-2 ELISA kit	R&D System	DY452
Human IL-1β ELISA kit	R&D System	DY201
Human IL-6 ELISA kit	R&D System	DY206
Human TNF-α ELISA kit	R&D System	DY210
Human IL-8 ELISA kit	R&D System	DY208
Substrate Reagent Pack	R&D System	DY999
Micro BCA Protein Assay Kit	Thermo Fisher Scientific	23235
SuperSignal West Femto Maximum Sensitivity Substrate	Thermo Fisher Scientific	34096
Maxima First Strand cDNA Synthesis Kit for RT-qPCR	Thermo Fisher Scientific	K1641
Radiant Green HiROX qPCR Kit	Alkali scientific	QS2050
ApopTag Fluorescein *In Situ* Apoptosis Detection kit	Sigma-Aldrich	S7110

**Deposited Data**		

Whole genome Microarray	NIH Gene Expression Omnibus	GSE200443

**Experimental Models: Organisms/Strains**		

Mouse: C57BL/6J	The Jackson Laboratory	https://www.jax.org/strain/000664

**Software and Algorithms**		

Prism 9.3.1	GraphPad	https://www.graphpad.com
GeneMANIA 3.3.5	Cytoscape 3.9.0	https://apps.cytoscape.org/apps/genemania

### Resource availability

#### Lead contact

Further information and requests for resources and reagents should be directed to and will be fulfilled by the lead contact, Ashok Kumar (akuma@med.wayne.edu).

#### Materials availability

This study did not generate new unique reagents.

### Experimental model and subject details

#### Bacterial strains

The bacterial strains used in this study are *Staphylococcus aureus* RN6390 (MSSA) and *Staphylococcus aureus* USA300 (MRSA). The strains were routinely cultured in Tryptic Soy medium (TSA or TSB; Sigma, St. Louis, MO) at 37°C.

#### Mice and ethics statement

Both male and female C57BL/6 (B6) mice (age, 6-8 weeks) were purchased from the Jackson Laboratory (Bar Harbor, ME, USA) and were housed in a restricted access DLAR facility at the Kresge Eye Institute, maintained in a 12:12 light/dark cycle, and fed rodent chow (Labdiet; Pico Laboratory, St. Louis, MO, USA) and water *ad libitum*. Mice were used and treated in compliance with the Association for Research in Vision and Ophthalmology (ARVO) Statement for the Use of Animals in Ophthalmic and Vision Research. All procedures were approved by the Institutional Animal Care and Use Committee (IACUC) of Wayne State University under protocol #IACUC-19-03-1012.

#### Mouse model of bacterial endophthalmitis

Bacterial endophthalmitis was induced in B6 mice (both sexes and 6-8 weeks old) by intravitreal injection with specified doses of bacteria as described previously (Singh et al., 2020). As per our IACUC approved protocol, only one eye of each mouse can be injected with either sterile PBS (serving as control) or bacteria. Overnight-grown cultures were rinsed and diluted in 1X PBS accordingly to obtain 5000 cfu inoculum per eye. Mice were anesthetized with ketamine and xylazine and intravitreal injections of PBS or bacteria (2μl volume) were performed using 34-gauge needle under a microscope. This procedure is routinely performed in the lab and reported in our several studies (Kumar et al., 2010, 2016; Talreja et al., 2014a). For treatment groups (DC: 10μg, CT: 10μg, Glb: 10μg, vancomycin: 0.7μg) were administered similarly via the intravitreal route in 1μL volume either 12h prior to infection or 6h post-infection.

#### Cell and culture conditions

Human retinal pigment epithelial cell line, ARPE-19, was maintained in Dulbecco’s modified Eagle’s medium Nutrient mixture F-12 (DMEM F-12) whereas, human Müller glial cell line, MIO-M1, was cultured in DMEM GlutaMAX, both with supplementation of 10% Fetal Bovine Serum (FBS) and 1% Penicillin-Streptomycin antibiotic solution and 10 μg/mL L-glutamine at 37 °C in 5% CO_2_. However, the cells were cultured in antibiotic and serum-free media overnight before infection, followed by 1h drug pretreatment and infection with *S. aureus* (MOI 10:1) for 6h.

### Method details

#### Bacterial infection and drugs treatment

For *in vitro* and *in vivo* infection experiments, overnight-grown bacteria were rinsed and diluted in 1X PBS to reach the desired Colony Forming Units (CFU). To make heat-killed bacteria, the prepared inoculum in PBS was subjected to 90 °C heat treatment using a dry bath for 10 min, cooled down, and used. The non-viability of the bacteria was confirmed by plating on TSA plate. The three drugs used in the study: Dequalinium chloride (sc-214869), Clofilium tosylate (sc-391228) and Glybenclamide or Glyburide (sc-200982) were purchased from Santa Cruz Biotechnology,Inc., Dallas, TX). For experiments involving LPS, 10 μg/mL of LPS was added to cell lines post drug treatment and incubated for 8h.

#### Cellular toxicity assay

Cell viability was determined using 3-(4,5-dimethylthiazol-2-yl)-2,5-diphenyltetrazolium bromide (MTT) assay (Invitrogen)(Singh et al., 2021a). Cells were seeded in DMEM in a 96-well plate overnight in a 37 °C incubator with 5% CO2. The cells were incubated with varying concentrations of DC, CT or Glb for 16h, followed by three washes with 1XPBS and supplemented with fresh DMEM. Next, MTT reagent (5 mg/mL in PBS) was added to each well and incubated for 4h at 37 °C. The supernatant was then aspirated, followed by the addition of 100μL of cell lysis buffer (20% SDS in 50% DMF) for an hour. The absorbance was measured using a microplate reader (Synergy multi-mode reader, BioTek, Winooski, VT, USA) and the cell viability was expressed as a percentage over control and calculated using the formula (mean OD of treated cells/mean OD of untreated control cells) X 100 and expressed as cell viability (%).

#### RNA extraction, cDNA synthesis and qPCR

Total RNA was extracted from cultured retinal cells or mouse retinas using TRIzol reagent, as per the manufacturer’s protocol (Invitrogen, Carlsbad, CA). Next, cDNA was synthesized using 1 μg of the isolated RNA using a Maxima first strand cDNA synthesis kit, according to manufacturer’s instructions (Thermo scientific, Rockford, IL). The cDNA was then subjected to qRT-PCR on a StepOnePlus Real-Time PCR System (Applied Biosystems, Foste City, CA, USA) using gene-specific PCR primers from Integrated DNA Technologies (Coralville, IA, USA) with a PCR condition of initial denaturation at 94 °C for 5 min, followed by 40 cycles of denaturation (94 °C, 45 s), annealing (60 °C, 1 min), and extension (72 °C, 45 s), with a final extension at 72 °C for 10 min. The data were analyzed as a comparative ΔΔC_T_ method and were presented corresponding to the fold-change differences in gene expression in test samples with respect to control.

#### Cytokine ELISA

Following infection, the cell-free culture supernatants from *in vitro* experiments were collected and the levels of IL-1β, IL-6, IL-8, TNFα, CXCL1 and CXCL2 were determined by ELISA using commercially available kits as per manufacturer’s instructions [R & D systems, Minneapolis, MN]. For *in vivo* cytokine estimation, whole eyes from mice were enucleated, homogenized in 1X PBS by beating against stainless steel beads using a Tissue lyser (Qiagen, Valencia, CA, USA), centrifuged and the supernatants were subjected to ELISA as already mentioned. Importantly, prior to performing ELISA, samples were quantified using BCA method ensuring equal protein concentrations.

#### Minimum inhibitory concentration (MIC) determination

A micro broth-dilution method was used to determine the MIC (Singh et al., 2021b) for dequalinium chloride, clofilium tosylate, and gluburide. Briefly, bacterial cultures (10^5^ CFU/well) were exposed to a two-fold serial dilution of the test compound in a 96-well plate. Following overnight incubation, the optical density (A_600_) of each microplate well was recorded using a spectrophotometer. MICs were determined based on the optical density of the growth in control and the lowest drug concentrations that resulted in *S. aureus* growth inhibition compared with media alone.

#### Bacterial burden determination

Bacterial densities in infected eyes of WT mice were assessed using the standard serial dilution and bacterial plate count method. The eyes were enucleated and homogenized in sterile 1X PBS in a Tissue lyser (Qiagen, Valencia, CA, USA), at the indicated time points, followed by serial dilution and plating on tryptic soy agar (TSA) plates. Results were expressed as mean ± SD number of colony-forming units (cfu)/eye.

#### Retinal function testing

Scotopic electroretinogram was done to evaluate retinal function in *S. aureus* induced endophthalmitis (Singh et al., 2020) (Francis et al., 2020). ERGs were recorded following bilateral mydriasis and dark overnight adaptation using the Celeris ERG system (Diagnosis LLC, Lowell, MA, USA) according to the manufacturer’s instructions. The ERG a-wave was measured as an amplitude between the ERG baseline and the first negative peak, and the ERG b-wave was measured as an amplitude between the first negative peak and the first positive peak. Data were analyzed with respect to placebo control retinas.

#### TUNEL assay

To determine retinal cell death, the eyes were fixed in Tissue-Tek OCT (Sakura, Torrance, CA, USA) and 5μm thick cryosections were collected from each eye and mounted onto microscope slides. TUNEL staining was performed on the sections using ApopTag fluorescein *in situ* apoptosis detection kit according to the manufacturer’s instruction (Millipore, Billerica, MA, USA).

#### Immunoblotting

Following infection, two retinas were pooled in RIPA buffer, sonicated and lysates were obtained after centrifugation. The lysed samples were quantified using Micro BCA protein assay kit (Thermo Scientific, Rockford, IL) and subsequently run on SDS polyacrylamide gels, electro-transferred to 0.45μm nitrocellulose membrane using a wet blot transfer. The membrane was then treated with 5% skim milk in TBST (20 mM Tris HCl [pH 7.6], 0.15 M sodium chloride, and 0.5% Tween 20), for 1h at RT and further incubated with respective 1° antibodies (Cell Signaling, USA or SantaCruz Biotechnology, USA) in 3% BSA in TBST (1:1000 dilution) for overnight on a rocker at 4°C. After wash, the membrane was further treated with horseradish peroxidase conjugated appropriate 2° antibodies (anti-mouse or anti-rabbit Ig) for 2h. Following three washes, the blots were developed with the enhanced chemiluminescence kit.

#### Transcriptome analyses, CMAP and network analysis

Genome level transcriptome analysis of *SA* endophthalmitis was performed to identify potential target for intervention. The transcriptome profiling of *SA*-infected vs. uninfected B_6_ mouse retina showed significant temporal expression changes in 1,234 genes. From the gene expression profiles of *SA* infected samples, a signature of the top 200 upregulated and downregulated genes was developed that distinguished the infected samples from uninfected controls. This signature was used to identify individual small molecules/drugs or their combinations from the C-Map 2.0 database that anti-correlated with the infection signature, i.e. those which counter-regulate the infection-perturbed genes and/or pathways. The top three drugs against *SA* infection were identified that induced maximum counter-regulation and minimum co-regulation of infection signature genes (Lamb et al., 2006). For the network analysis, the top differentially expressed genes from the drug signatures were analyzed using the GeneMANIA [version: 3.3.5] plugin in Cytoscape [Version: 3.9.0] and the gene interaction networks were built. The interaction networks for the genes were based on co-expression, genetic interaction, pathways, physical interactions, and attributes from databases like MSigdb, NCI Nature, etc. The top 20 related genes and at most 20 attributes based on the default weighting was used to generate the network. The hubs of the interaction network were the nodes with the top 20 percentile degree value and the bottlenecks were the nodes with top 20 percentile betweenness centrality value. The upregulated genes were analyzed for enrichment using the Metascape tool (Zhou et al., 2019).

### Quantification and statistical analysis

All the assays were performed independently three times in biological triplicates and graphs were plotted showing mean ± standard deviation. The data were analyzed using Graph Pad Prism version 9.3.1 (Graph Pad, San Diego, CA). Statistical significances were determined using ANOVA with multiple comparisons as indicated in the figure legends. A confidence interval of 95% was maintained for all experimental values. A p value < 0.05 was considered statistically significant.

## Data Availability

The data generated from this study is deposited in the NIH Gene Expression Omnibus. The accession id for the data is GSE200443.
