# Supplementary material for: Transcriptomics and systems biology identify non-antibiotic drugs for the treatment of ocular bacterial infection
Source: iScience. 2022 Aug 2;25(9):104862. doi: 10.1016/j.isci.2022.104862 (PMC9399287; doi:10.1016/j.isci.2022.104862)
Supplement: Document S1. Figures S1–S5 [file mmc1.pdf]

iScience, Volume *25*

Supplemental information

Transcriptomics and systems biology identify non-antibiotic drugs for the treatment of ocular bacterial infection

Susmita Das, Sukhvinder Singh, Sarthak Satpathy, Manoj Bhasin, and Ashok Kumar

Supplementary Data:

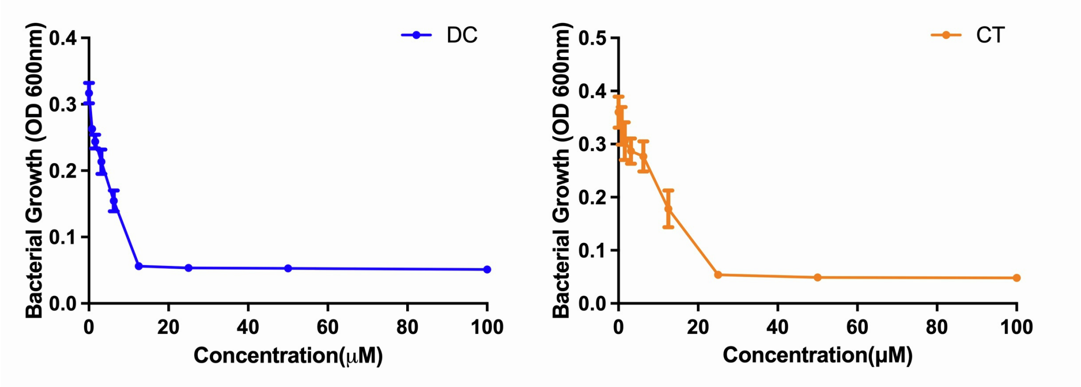

Figure S1. Determination of minimum inhibitory concentration (MIC) of Dequalinium chloride (DC) and Clofilium tosylate (CT) in Müller Hinton broth (MHI) against *Staphylococcus aureus* RN6390, Related to Figure 3. The values shown represent the combined results of two separate experiments. Values are the mean of the three replicates for each drug concentration used.

(A)

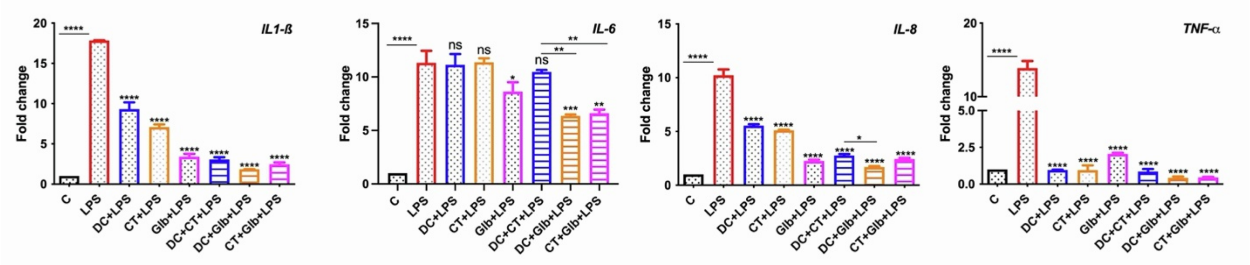

(B)

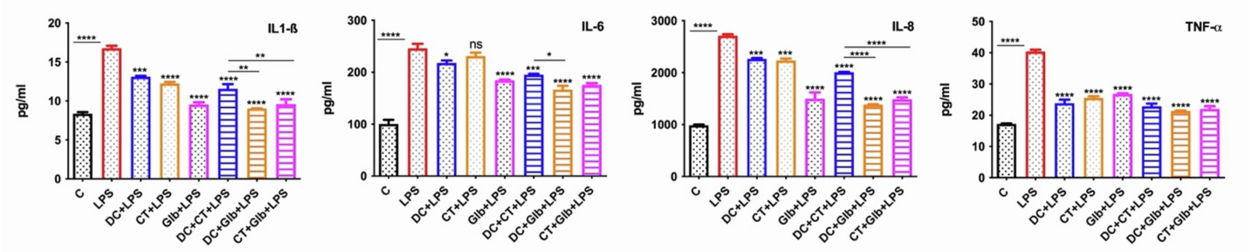

Figure S2. Assessment effect of DC, CT and Glb on inflammatory responses in cultured human Müller glial cells in response to LPS stimuli, Related to Figure 3. Human retinal Müller glial cells (MIO-M1) were treated with DC (25μM), CT (25μM) and Glb (50μM) for 1 hour, followed by 10μg/ml of LPS stimulation for 8h. Cells were then harvested for qPCR analysis of inflammatory cytokines **(A)** and culture supernatants were used to quantify the protein levels via ELISA **(B).** Statistical analysis was performed using one way ANOVA (*) p<0.05 (**) p<0.01 (***) p<0.001 (****) p<0.0001. Significance was compared between control (C) vs SA samples and SA vs drug-treated samples,.

(A)

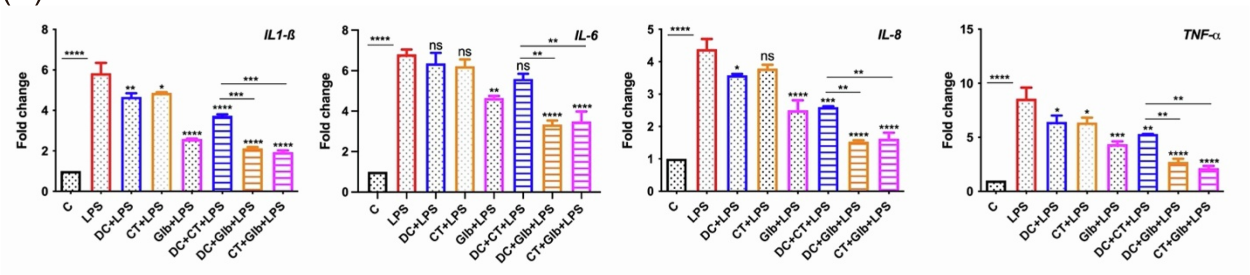

(B)

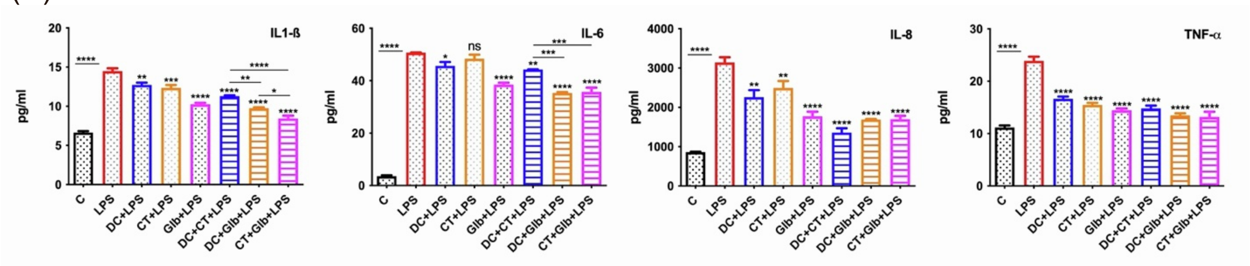

Figure S3. Assessment of DC, CT and Glb on inflammatory responses in cultured human retinal pigment epithelial cells in response to LPS stimuli, Related to Figure 4. Human retinal RPE cells (ARPE-19) were treated with DC (25μM), CT (25μM) and Glb (50μM) for 1 hour, followed by 10μg/ml of LPS stimulation for 8h. Cells were then harvested for qPCR analysis of inflammatory cytokines **(A)** and culture supernatants were used to quantify the protein levels via ELISA **(B).** Statistical analysis was performed using ANOVA (*) $p<0.05$ (**) $p<0.01$ (***) $p<0.001$ (****) $p<0.0001$, ns; non-significant. Significance was compared between control (C) vs SA samples and SA vs drug-treated samples.

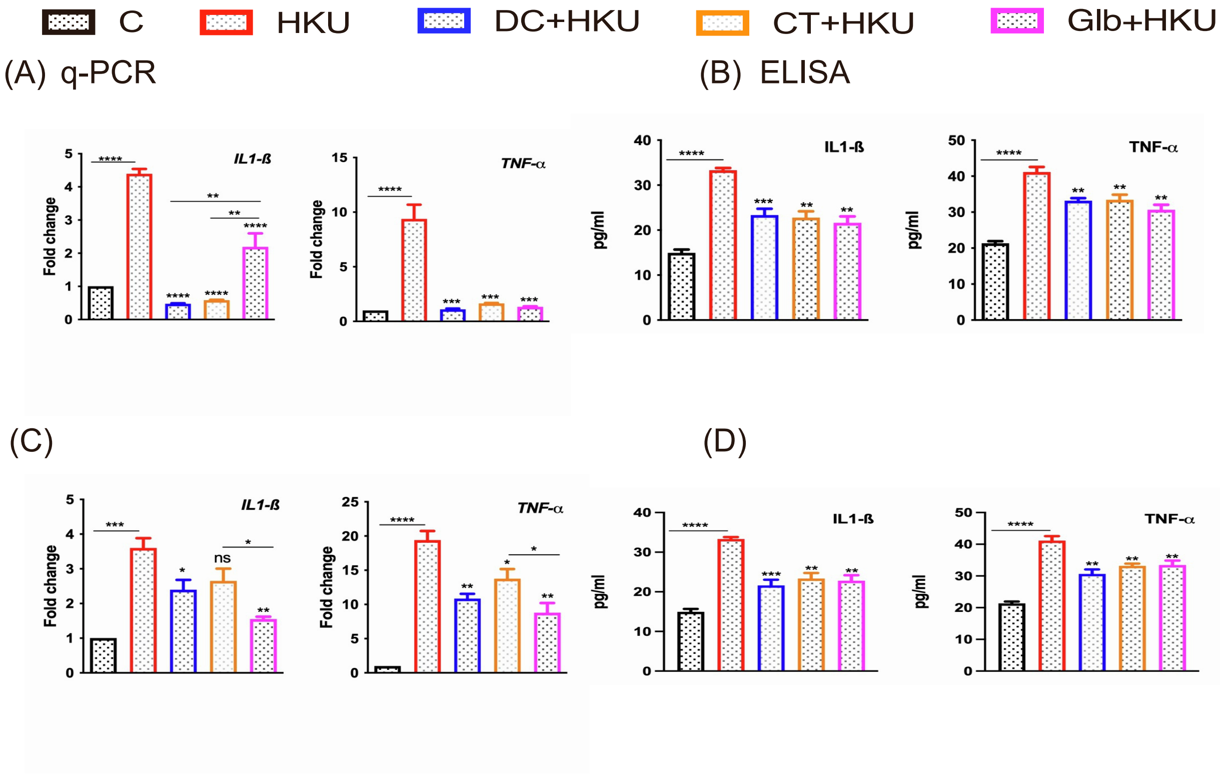

Figure S4. Effect of DC, CT, and Glb on inflammatory responses in cultured human retinal cells post infection with heat-killed *S. aureus* USA300, Related to Figure 5. **(A-B)** Human retinal muller glial cells (MIO-M1), and **(C-D)** human retinal pigment epithelial cells (ARPE-19 cells) were treated with DC (25μM), CT (25μM) and Glb (50μM) for 1 hour, followed by infection with heat-killed *S. aureus* USA300. 6 h p.i., cells were harvested for qPCR analysis of inflammatory cytokines **(A, C)** and culture supernatants were used to quantify the protein levels via ELISA **(B, D).** Statistical analysis was performed using ANOVA (*) $p<0.05$ (**) $p<0.01$ (***) $p<0.001$ (****) $p<0.0001$; ns, non-significant. Significance was compared between control (C) vs SA samples and SA vs drug-treated samples.

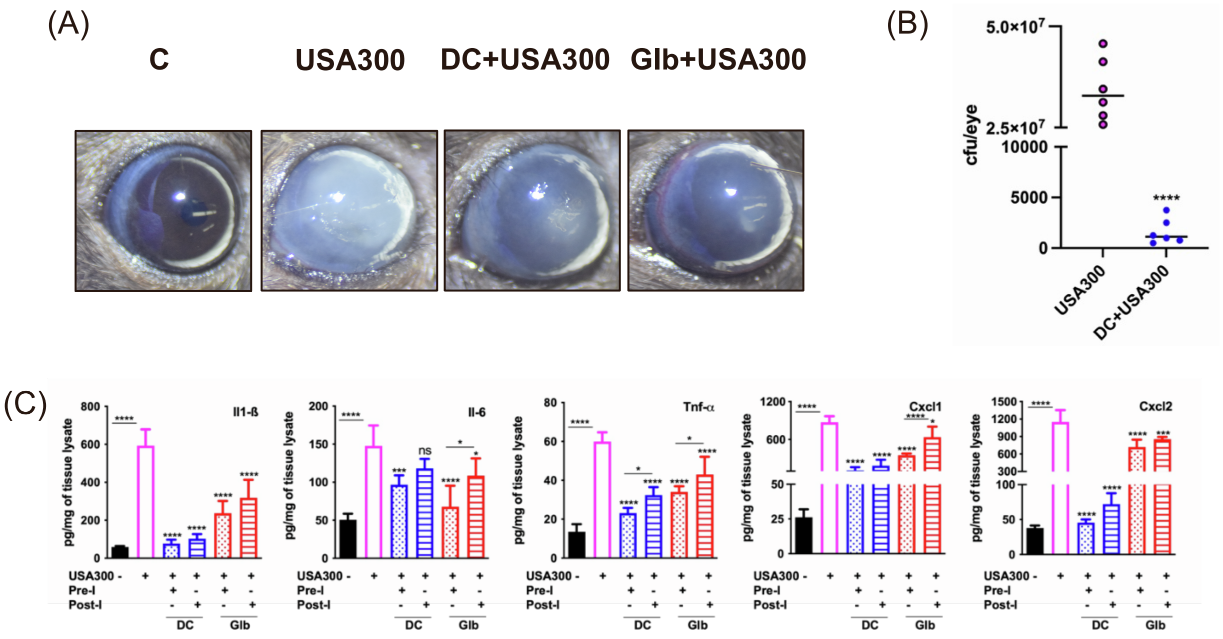

Figure S5. Prophylactic efficacy of the drugs against *S. aureus* USA300 induced endophthalmitis in mice, Related to Figure 6. C57BL/6 mice eyes (n = 6 eyes) were intravitreally injected with each drug (10μg per eye). After 12h post drug administration, 5000 colony forming units (cfu/eye) of *S. aureus* USA300 were injected intravitreally. PBS treated eyes served as control (C). Eyes were processed 24h post-infection. **(A)** Microscopic eye examination was performed, and photomicrographs were taken from representative eyes showing corneal haze/opacity. **(B)** At 24h p.i. eyes were enucleated, homogenized, and the bacterial burden was estimated via serial dilution plating. **(C)** The lysates from infected, drug-treated and control eyes were subjected to ELISA to quantify protein levels of indicated inflammatory mediators. Drug treatment, pre and post infection were labelled as Pre-I and Post-I respectively. Statistical analysis was performed using ANOVA (*) $p<0.05$ (***) $p<0.001$ (****) $p<0.0001$, ns; non-significant. Significance was compared between control (C) vs SA samples and SA vs drug-treated samples.
